# Newcastle disease virus matrix protein interacts with chicken eukaryotic elongation factor 2 to promote viral protein production and enhance viral particle yield

**DOI:** 10.1186/s13567-026-01876-x

**Published:** 2026-11-14

**Authors:** Jinyang Liu, Yingxu Ren, Jiaqi Chen, Dandan Long, Jiafu Zhao, Zenglei Hu, Zhiqiang Duan

**Affiliations:** 1 College of Animal Science, Guizhou University, Guiyang, 550025 China; 2 Key Laboratory of Animal Genetics, Breeding and Reproduction in The Plateau Mountainous Region, Ministry of Education, Guizhou University, Guiyang, 550025 China; 3 Joint International Research Laboratory of Agriculture and Agri-Product Safety, Ministry of Education, Yangzhou University, Yangzhou, 225009 China

**Keywords:** Newcastle disease virus, matrix protein, chicken, eukaryotic elongation factor 2

## Abstract

Eukaryotic elongation factor 2 (eEF2) is important for the step of translation elongation and protein synthesis, while viruses can exploit eEF2 to potentiate their own replication. Previously, chicken eEF2 was identified as a new binding partner with the Newcastle disease virus (NDV) matrix (M) protein, but their actual interactions and functions in the replication and pathogenicity of NDV are not completely understood. In this study, the NDV M protein was verified to directly interact with chicken eEF2 in the cytoplasm. In addition, four residues (Y187, R188, S197, and S199) in the N-terminus region (aa 179–234) of M protein and six residues (E753, Q754, G758, Y760, N764, and S802) in the V domain (aa 727–858) of chicken eEF2 were responsible for their binding. However, although six residues of eEF2 were highly conserved among human, other mammals, and avian species, four residues of the M protein were completely nonconservative between NDV and other paramyxoviruses. Moreover, mutation of key interactional residues (Y187A/R188A/S197A/S199A) in the M protein remarkably attenuated the virulence, replication ability, and pathogenicity of NDV. Furthermore, interaction of the M protein with chicken eEF2 promoted its expression at the translation level and reduced its phosphorylation level on Thr57 residue, which was conducive to the elevated viral protein production and viral budding and release, and thereby enhanced viral particle yield to facilitate the replication and pathogenicity of NDV. Collectively, these findings demonstrate the significance of M-eEF2 interaction in the replication and pathogenicity of NDV and offer a potential therapeutic target for developing novel anti-NDV strategies.

**Supplementary information:**

The online version contains supplementary material available at https://doi.org/10.1186/s13567-026-01876-x.

## Introduction

Newcastle disease (ND), caused by the infection of Newcastle disease virus (NDV), is a highly contagious disease that can cause substantial threat and is economically catastrophic to the global poultry industry [1]. As a typical representative of the *Paramyxoviridae* family, NDV contains a single-stranded, negative-sense, nonsegmented RNA genome of approximately 15.2 kb in length, which encodes six structural proteins (known as NP, P, M, F, HN, and L), as well as two nonstructural proteins (known as V and W) due to the RNA editing of viral *P* gene [2, 3]. Among these viral proteins, the NDV M protein is one of the most abundant proteins in virions, which constitutes a bridge between viral nucleocapsid and envelope and plays a crucial role in virions’ assembly and budding during virus infection [4, 5]. In addition, the NDV M protein is also demonstrated to be a nuclear-cytoplasmic shuttling protein that participates in the inhibition of host cell transcription, regulation of viral RNA synthesis, and transcription and promotion of viral immune evasion [6–8]. These functions of M protein are closely related to the virulence, replication efficiency, and pathogenicity of NDV [9], thus revealing the multifunctionality of M protein in the NDV life cycle.

It is well-recognized that the close interactions between viral proteins and host proteins are of great significance for viral infection and replication [10, 11], and each protein of NDV has been reported to regulate viral replication and pathogenicity by interacting with host proteins [12–14]. Regarding the NDV M protein, it has been shown that the interaction of M protein with host importin β1 or ANP32B mediates its nuclear localization or nuclear accumulation, which promotes viral RNA synthesis and transcription in the cytoplasm and thereby augments viral replication and pathogenicity [7, 15, 16]. Beyond that, the NDV M protein also interacts with host CHMP4B to promote viral budding efficiency and enhance viral virulence and pathogenicity [17, 18]. Remarkably, a recent study revealed that paramyxovirus M proteins can modulate host cell translation via interacting with core exon-junction complex (cEJC) in the cytoplasm, which enhances viral protein production and subsequent viral replication [19]. In our previous studies, we found that the NDV M protein indirectly increases the expression of ribosomal protein L18 (RPL18) and in turn promotes viral protein biosynthesis and viral replication, suggesting the potential function of NDV M protein in viral protein production [20]. However, there is currently no direct evidence to demonstrate that the NDV M protein interacts with host proteins to carry out this function.

Eukaryotic elongation factor 2 (eEF2) is an evolutionarily conserved phosphorylated protein that drives the GTP-dependent translocation of the nascent protein chain to participate in cellular mRNAs translation, but the increased phosphorylation of eEF2 on Thr57 residue [peEF2 (Thr57)] reduces its affinity for the ribosome, resulting in arresting the step of the translation elongation and protein synthesis [21, 22]. In recent years, an increasing number of studies have shown that viruses can hijack eEF2 to facilitate their own replication. For instance, cells infected with the alphaviruses, including Chikungunya virus (CHIKV), Venezuelan equine encephalitis virus (VEEV), and Sindbis virus (SINV), show a > 50-fold increase of the peEF2 (Thr57) level [23]. Meanwhile, the elevated peEF2 (Thr57) level is beneficial for the rapid shut-off of host translation to counter the interferon β-stimulated transcriptional response, thus enabling SINV to replicate more efficiently in mammalian cells [24]. On the contrary, coxsackievirus B3 infection leads to the decreased peEF2 (Thr57) level via multiple signaling cascades, which enhances virus replication through promoting the translation elongation and production of viral proteins [25]. In addition, other studies found that a variety of components of cellular translational machinery, such as eukaryotic initiation factor 2 alpha (eIF2α), eIF3b, eIF4E, and eEF2, are enriched in areas surrounding the viral factories that contribute to the increased viral protein synthesis in African swine fever virus-infected cells [26]. Moreover, it has been shown that host proteins interacting with bovine viral diarrhea virus (BVDV) mainly focus on translation, protein synthesis, and cellular metabolism pathways, of which eEF2 serves as one of the hub proteins associated with protein synthesis, indicating the importance of eEF2 in BVDV replication [27]. However, whether eEF2 and peEF2 (Thr57) are involved in the replication and pathogenicity of NDV is not known.

We previously found that chicken eEF2 is an interaction partner of the NDV M protein using yeast two-hybrid screening assay [15]. Similarly, the binding of NDV M protein to eEF2 is also found in other two research groups by using co-immunoprecipitation (Co-IP) combined with liquid chromatography–tandem mass spectrometry methods [28, 29]. Nevertheless, the biological significance of this binding in NDV replication and pathogenicity has not been fully characterized. In the present study, we demonstrate for the first time that the NDV M protein directly interacts with chicken eEF2 in the cytoplasm, which increases the eEF2 expression level and decreases the peEF2 (Thr57) level to promote viral protein production and enhance viral particle yield, thereby facilitating the replication and pathogenicity of NDV. These findings not only offer insights into the underlying mechanism of M–eEF2 interaction in NDV replication and pathogenicity but also provide a novel target for antiviral treatment strategies against NDV.

## Materials and methods

### Cells, viruses, chemical compounds, and antibodies

Chicken embryo fibroblast cell lines (DF-1) were a kind gift from Prof. Xiufan Liu (Yangzhou University, China), which were cultured in Dulbecco’s modified Eagle medium (DMEM) containing 15% fetal bovine serum (FBS) (Gibco, USA) at 37 °C in an atmosphere with 5% CO_2_. The virulent NDV strain SS1 (Sheldrake duck/China/Guizhou/SS1/2014; GenBank no. KP742770.1) and its recombinant virus rSS1RFP expressing the red fluorescent protein (DsRed-Express) gene were preserved in our laboratory [30]. The two viruses were propagated once in 9-day-old embryonated specific pathogen-free (SPF) chicken eggs and then stored at −80 °C until use. The chemical compounds NH125 and A-484954 were purchased from MedChemExpress (USA). The antibodies used in this study were as follows: mouse anti-NDV M and NP (prepared in our laboratory), mouse anti-NDV HN (Santa Cruz Biotechnology, USA), rabbit anti-eEF2 and anti-peEF2(Thr57) (Affinity Biosciences, USA), mouse anti-HA, anti-His, anti-GST, anti-GAPDH, rabbit anti-Myc, and FITC-labeled goat anti-mouse or Cy3-labeled goat anti-rabbit IgG (H+L) (secondary antibody) (Beyotime Biotech, China).

### Plasmid construction

The plasmids pCMV-HA, pCMV-Myc, pET-32a (+), and pGEX-6p-1 were purchased from Clontech (USA), Novagen (USA), and GE Healthcare (USA), respectively. The coding sequences of NDV *M* gene (GenBank no. KP742770.1) and chicken *eEF2* gene (GenBank no. NM_205368.2) containing appropriate restriction sites were synthesized by Tsingke Biotechnology Co., Ltd. (Beijing, China), and then inserted into the corresponding empty vectors to generate pCMV-HA-M, pCMV-Myc-eEF2, pGEX-6p-M, and pET-32-eEF2, respectively. In addition, all kinds of the truncated mutants, deleted mutants, or point mutation mutants of NDV M protein and chicken eEF2 expressed in the plasmids were generated as described above. All the constructed recombinant plasmids were confirmed by restriction enzyme digestion and DNA sequencing.

### Indirect immunofluorescence assay

DF-1 cells cultured on 14-mm coverslips were infected with 0.1 multiplicity of infection (MOI) SS1 at 37 ℃. At 12- and 24-hours post-infection (hpi), the cells were washed with precooled phosphate buffer saline (PBS), fixed with 4% paraformaldehyde for 20 min, permeabilized with 0.2% Triton X-100 for 5 min, and then blocked with 10% FBS in PBS for 30 min. After that, the cells were incubated with the mouse anti-M and rabbit anti-eEF2 antibodies diluted in PBS containing 10% FBS at 37 °C for 1 h. After three washes with PBS, the cells were incubated with FITC-labeled goat anti-mouse and Cy3-labeled goat anti-rabbit IgG (H+L) antibodies at 37 °C for 1 h. Thereafter, cells were counterstained with DAPI (Beyotime Biotech, China) to detect nuclei, and the coverslips were mounted with PBS containing 50% glycerol. Images were captured with a laser confocal scanning microscope (Nikon, Japan), and the line intensity profile of proteins was analyzed using ImageJ software (National Institutes of Health, USA).

### Protein–protein interaction assays

For Co-IP assays, DF-1 cells cultured in 35-mm dishes were cotransfected with the indicated plasmids co-expressing the M protein and eEF2 or their mutant proteins. At 24 h post-transfection (hpt), cells were washed three times with PBS and then lysed with 1 × RIPA lysis buffer (Beyotime Biotech, China). The lysis supernatants were collected and then incubated with mouse anti-HA or rabbit anti-Myc antibody overnight at 4 °C. The complexes were recovered by adsorption to protein A+G Agarose (Beyotime Biotech, China) at room temperature for 1 h. After three washes with RIPA lysis buffer, the coimmunoprecipitated products were detected by western blot using rabbit anti-Myc or mouse anti-HA antibody.

For pull-down assays, the His-eEF2 fusion protein (6 h of induction with 0.2 mM IPTG at 20 °C) and GST-M fusion protein (4 h of induction with 0.5 mM IPTG at 28 °C) were first expressed in *E. coli* BL21 (DE3), and the soluble His-eEF2 and GST-M proteins were purified using His-Tag or GST-Tag Protein Purification Kit with Magnetic Agarose Beads (Beyotime Biotech, China), respectively. In the GST pull-down assay, the GST-M or GST tag bound to Anti-GST Magnetic Beads (Beyotime Biotech, China) were incubated with His-eEF2 for 2 h at 4 °C. After washing three times with PBS, the protein complexes were separated by boiling and then used for SDS-PAGE followed by western blot analysis. In the His pull-down assay, anti-His Magnetic Beads-bound His-eEF2 or His-Tag was incubated with GST-M for 2 h at 4 °C. The beads were treated as described above, and the target protein was detected by western blot.

### Western blot analysis

The proteins derived from plasmids-transfected or NDV-infected cells were obtained at the indicated time points and then quantitated using a bicinchoninic acid kit (Beyotime Biotechnology, China) to standardize the contents. The protein samples were processed, separated using 10% SDS-PAGE, and then transferred to polyvinylidene difluoride (PVDF) membrane followed by blocking with 10% fat-free milk for 2 h at room temperature. Subsequently, the PVDF membranes were incubated with the diluted primary and secondary antibodies, respectively, at 37 °C for 1 h, and the protein bands were observed using an enhanced chemiluminescence (ECL) detection system (Bio-Rad, USA). The expression of GAPDH in protein samples was used to normalize the difference of detected target protein levels, and the grayscale values of the protein bands were determined with ImageJ software.

### Molecular docking and multiple sequence alignment

The crystal structures of NDV M protein [Protein Data Bank (PDB) accession no. 4G1G] and chicken eEF2 (PDB accession no. 8Q87) were downloaded from the Research Collaboratory for Structural Bioinformatics (RCSB) Protein Data Bank (PDB). The interactions and binding sites between M protein and chicken eEF2 were predicted using AlphaFold3 via the AlphaFold Server. PyMOL 2.5.5 software (Schrödinger, USA) was used to generate the structural annotations. As for multiple sequence alignment, the amino acid (aa) sequences of NDV and other paramyxovirus M proteins or eEF2 from humans and other selected species were retrieved from the GenBank database, which were then analyzed using the Clustal W multiple alignment algorithm in the MegAlign program (DNASTAR Inc., USA). The aa conservation of the M protein and eEF2 was obtained based on the NDV M–chicken eEF2 interaction sites and the multiple sequence alignment results.

### Reverse genetics rescue of the mutant NDV

The full-length cDNA clone pNDV/SS1RFP-M/4M harboring four alanine (A) substitutions in the M protein (Y187A/R188A/S197A/S199A) was constructed by the methods of overlapping polymerase chain reaction (PCR) (primers presented in Table 1), enzymatic digestion, and ligation (Additional file 1). For rescue of the mutant virus, 1 μg of pNDV/SS1RFP-M/4M together with three helper plasmids (pCI-NP with 1 μg, pCI-P with 0.5 μg, and pCI-L with 0.5 μg) were cotransfected into BSR-T7/5 cells. The supernatants and cell monolayers were collected at 60 hpt and then mixed and inoculated into the allantoic cavity of 9-day-old embryonated SPF chicken eggs. The hemagglutination (HA) titer test and DNA sequencing were performed to identify the rescued virus rSS1RFP-M/4M at 3 days after inoculation. In addition, rSS1RFP-M/4M was passed through successive generations (up to five times) in SPF embryonated eggs to evaluate its stability using HA test and DNA sequencing. Moreover, the biological characteristics [including mean death time (MDT), intracerebral pathogenicity index (ICPI) value, egg infectious dose (EID)_50_, and tissue culture infective dose (TCID)_50_] and in vitro growth kinetics of the parental (rSS1RFP) and mutant (rSS1RFP-M/4M) NDVs were determined as described previously [6].

**Table 1:** **Primers used for plasmid construction or quantitative real-time (qRT)-PCR**

Primer name	Sequence (5′→3′)^*^	Product size (bp)	GenBank no.	Application
M/4M-F	ACA***ACCGGT***GCAGCACCCCTCTGAATTG	1731	KP742770.1	Plasmid construction
M/4M-a	CGCGCCCGCCACTTTCAATACTGCAGTTGGG ATCGCCGCGACATCCCTTCTCGGCACCACAGT			
M/4M-b	GCGGCGATCCCAACTGCAGTATTGAAAGTGG CGGGCGCGAACCTGTACAATCTTGCGCTCAAT	858		
M/4M-R	GTG***GTATAC***ATTGACTGCCTTATCTCCTG			
qNDV/M-F	CTGTGCTTGTGAAGGCGAGAGGT	100	KP742770.1	qRT-PCR
qNDV/M-R	TGGGGAGAGGCATTTGCTATAGGAT			
qDsRed-F	TCCTGTCCCCCCAGTTCCAGTA	124	KM486853.1	qRT-PCR
qDsRed-R	TCCTCGAAGTTCATCACGCGC			
qeEF2-F	GGCCGTGTTTTCTCTGGTCTCGT	114	NM_205368.2	qRT-PCR
qeEF2-R	GATGGTCCTTTGGATTGGCTTCA			
qGAPDH-F	TCAAGGCTGAGAACGGGAAACTTG	117	NM_204305.2	qRT-PCR
qGAPDH-R	TGGACTCCACAACATACTCAGCACC			

^*^The restriction sites (*Bsh*TI and *Bst*1107I) used in the primers M/4M-F and M/4M-R are shown in bold italic. The nucleotides shown in underline are the mutated sequences introduced in the primer to change the indicated residues to alanine (A).

### Pathogenicity assessment of the mutant NDV

The pathogenicity assessment of the mutant virus rSS1RFP-M/4 M and parental virus rSS1RFP was performed in 4-week-old SPF chickens. Briefly, 39 SPF chickens were randomly assigned into three groups, including rSS1RFP-M/4 M- (*n* = 13), rSS1RFP- (*n* = 13), and mock- (PBS; *n* = 13) infected groups. For each group, three birds and ten birds were used for sampling and clinical observation, respectively. Chickens inoculated with each virus at a dose of 10^5.0^ EID_50_/100 μL per bird or with 100 μL PBS were monitored for mortality rate statistics daily for 10 days. Three birds were euthanized at 4 days post-infection (dpi) for tissue sample collection and viral titer determination, and the part of tissues, such as spleen, thymus, bursa of Fabricius, and lung, was used for preparing pathological sections and conducting histopathological observations.

### Quantification of viral RNA synthesis and transcription

Viral RNA (vRNA) synthesis and transcription levels of the parental and mutant NDVs were measured by quantitative real-time PCR (qRT-PCR) assay at 6, 12, and 24 hpi, respectively. Primers qDsRed-F/qDsRed-R and qNDV/M-F/qNDV/M-R were used to detect the mRNA levels of the *DsRed* gene and viral *M* gene (Table 1), and the other primers for detecting vRNA synthesis (viral *N* and *P* genes) referred to the published articles [31]. qRT-PCR was performed using a SYBR Premix Ex Taq Kit (Takara, China) in Bio-Rad CFX-96 real-time fluorescence quantitative PCR instrument. The qRT-PCR mixture system and cycling parameters were performed as in previous studies [32]. The threshold cycle 2^−∆∆CT^ method was carried out to calculate the relative abundance of vRNA and mRNA levels, and the relative expression levels were normalized to those of the *GAPDH* gene.

### RNA interference of chicken eEF2 and virus infection

Three pairs of siRNAs (Table 2) targeting the chicken *eEF2* gene were designed to knock down its expression in DF-1 cells and evaluate its role in the replication and cytopathogenicity of NDV. As for the evaluation of the *eEF2* siRNA interference effect, low-passage DF-1 cells were transfected with the *eEF2* siRNAs and control siRNA using Lipofectamine^TM^ RNAiMAX transfection reagent (Thermo Fisher Scientific Inc., USA) according to the manufacturer’s instructions. At 48 hpt, the knockdown efficiency of *eEF2* siRNAs was validated by western blot. To investigate the effect of chicken *eEF2* knockdown on the replication and cytopathogenicity of NDV, eEF2 siRNA#2- or control siRNA-transfected DF-1 cells or normal control cells were infected with rSS1RFP at an MOI of 0.1. The vRNA synthesis, transcription, and translation of rSS1RFP were detected by qRT-PCR and western blot, respectively, at 6, 12, and 24 hpi. In addition, the cell culture supernatants collected at the indicated times were used to examine the virus titers. Meanwhile, the cytopathic effect (CPE) and DsRed expression were observed using an inverted fluorescent microscope (Nikon, Japan).

**Table 2:** **The information of chicken eEF2 siRNAsThe information of chicken eEF2 siRNAs**

Name	siRNA name	Sequence (5′→3′)	
Chicken eEF2 (GenBank no. NM_205368.2)	siRNA#1	Sense	GAUCGAUCCUGUGCUUGGUACAGUU
		Anti-sense	AACUGUACCAAGCACAGGAUCGAUC
	siRNA#2	Sense	CAGCUCAUCCUCGAUCCCAUCUUCA
		Anti-sense	UGAAGAUGGGAUCGAGGAUGAGCUG
	siRNA#3	Sense	CAGGAACGGUGCAUCACCAUCAAAU
		Anti-sense	AUUUGAUGGUGAUGCACCGUUCCUG
Control	Negative siRNA	Sense	UUCUCCGAACGUGUCACGUTT
		Anti-sense	ACGUGACACGUUCGGAGAATT

### Overexpression of chicken eEF2 and virus infection

To further explore the effect of chicken eEF2 overexpression on the replication and cytopathogenicity of NDV, low-passage DF-1 cells were transfected with pCMV-Myc or pCMV-Myc-eEF2, and the expression of Myc tag or Myc-eEF2 fusion protein was examined by indirect immunofluorescence assay at 24 hpt. As described above, pCMV-Myc- or pCMV-Myc-eEF2-transfected DF-1 cells or normal control cells were infected with rSS1RFP, and then vRNA synthesis, transcription, and translation of rSS1RFP, as well as virus titers, CPE, and DsRed expression were detected.

### Virion purification and viral budding efficiency characterization

DF-1 cells were transfected with the indicated siRNAs or plasmids and then infected rSS1RFP as described above. Virions were collected in the cell culture supernatants at 12 and 24 h after virus infection and purified as described previously [33]. The resulting virions were collected from the bottom and then dissolved in 100 μL of Tris–EDTA buffer solution. Total cellular proteins derived from the corresponding cell pellets were extracted by using a Universal Total Protein Extraction Kit with Mild Lysis Buffer and Spin Column (Beyotime Biotechnology, China). After the quantification of proteins, the viral HN and M proteins in the virions and whole cell lysates (WCLs) were then detected and quantitated as described previously [18].

### Chemical compounds treatment and virus infection

Different concentrations of chemical compounds NH125 (0, 2, 4, 8, and 12 μM) and A-484954 (0, 20, 40, 80, and 120 μM) were added to DF-1 cells; the CCK-8 and Ki67 assays were used to evaluate their effects to cell viability. In addition, the effect of NH125 (8 μM) and A-484954 (80 μM) on the expression levels of eEF2 and peEF2 (Thr57) were examined by western blot. Moreover, DF-1 cells infected with rSS1RFP or rSS1RFP-M/4M at an MOI of 0.1 were dealt with NH125 (8 μM) and A-484954 (80 μM), respectively. The vRNA synthesis, transcription, and translation, as well as viral budding efficiency of rSS1RFP or rSS1RFP-M/4M were detected by qRT-PCR and western blot, respectively, at the indicated time points. Meanwhile, the cell culture supernatants were collected and used to determine the viral titers.

### Statistical analysis

All experiments were repeated at least three independent times, and the data are expressed as mean ± standard deviation (SD). The statistical significance of the data was assessed by one-way or two-way analysis of variance with the GraphPad Prism 9.5 software (San Diego, USA). The differences are indicated by asterisks (^*^): ^*^P < 0.05, ^**^P < 0.01, and ^***^P < 0.001; no asterisk indicates no significant difference.

## Results

### NDV M protein interacts with chicken eEF2 in vitro and in vivo

**Figure 1:**
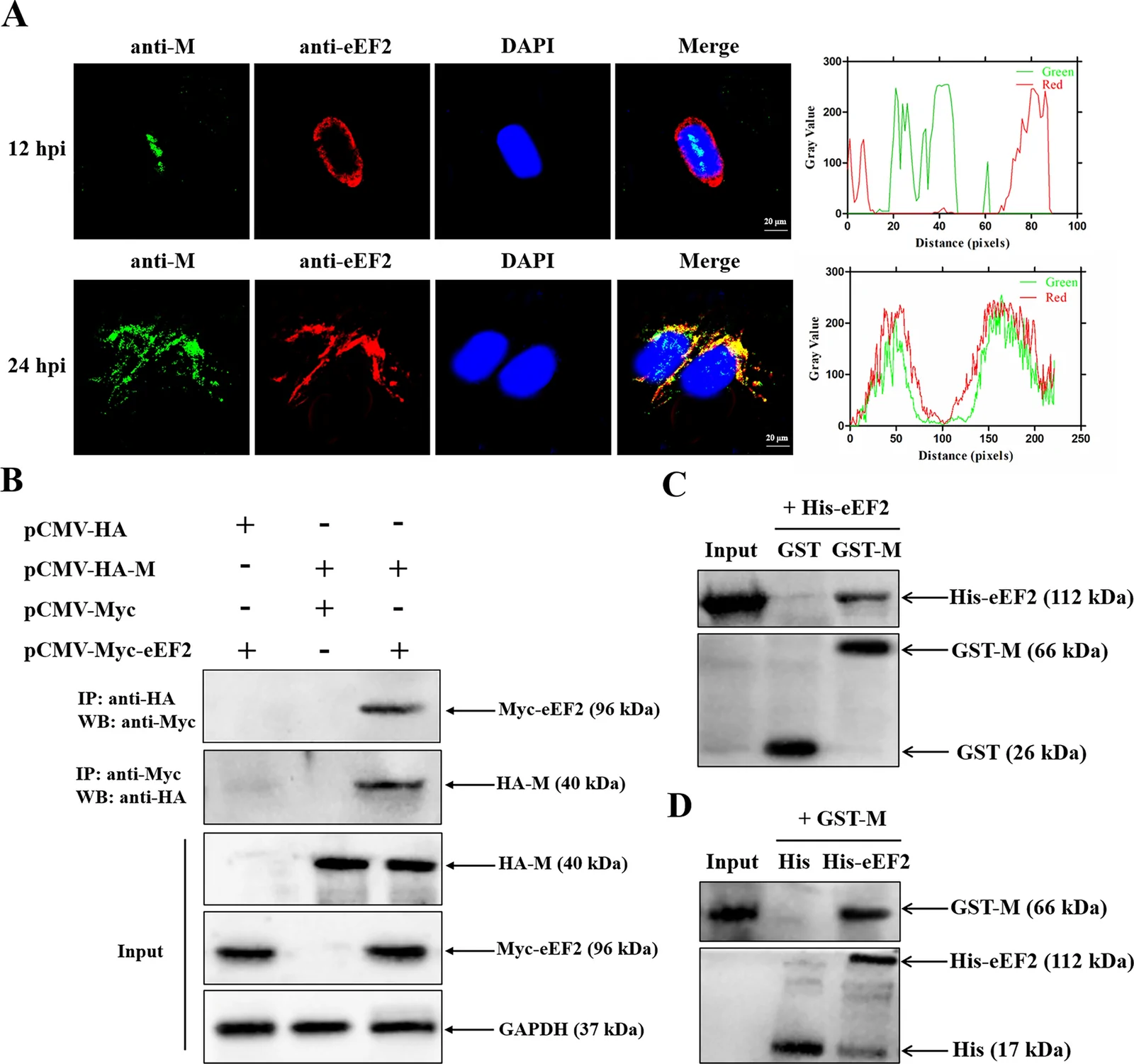
**Characterization of the interaction between NDV M protein and chicken eEF2.** (**A**) Fluorescence co-localization of M protein and chicken eEF2 in NDV-infected DF-1 cells using indirect immunofluorescence assay. The original magnification was 1 × 400. (**B**) Identification of the interaction between the M protein and chicken eEF2 in plasmids cotransfected DF-1 cells by Co-IP assay. Characterization of the interaction between the M protein and chicken eEF2 by GST pull-down assay (**C**) and His pull-down assay (**D**), respectively.

The fluorescence co-localization, Co-IP, and pull-down assays were performed to verify the interaction between NDV M protein and chicken eEF2. As shown in Figure 1A, the NDV M protein and chicken eEF2 were mainly localized in the nucleus and cytoplasm at 12 hpi, respectively, while most of the M protein had obvious co-localization with chicken eEF2 in the cytoplasm except for a few localizations in the nucleus at 24 hpi. In addition, the results of Co-IP assays showed that the HA-M fusion protein but not HA tag could be immunoprecipitated with Myc-eEF2 when using an anti-Myc antibody, and vice versa (Figure 1B). Moreover, an in vitro pull-down binding assay revealed that the His-eEF2 fusion protein could be pulled down by the GST-M fusion protein but not by the GST tag (Figure 1C), and in turn, the GST-M fusion protein was also pulled down by the His-eEF2 fusion protein rather than by the His tag (Figure 1D). These results suggested that the NDV M protein directly interacted with chicken eEF2 in the cytoplasm.

### Mapping the binding regions between NDV M protein and chicken eEF2

**Figure 2:**
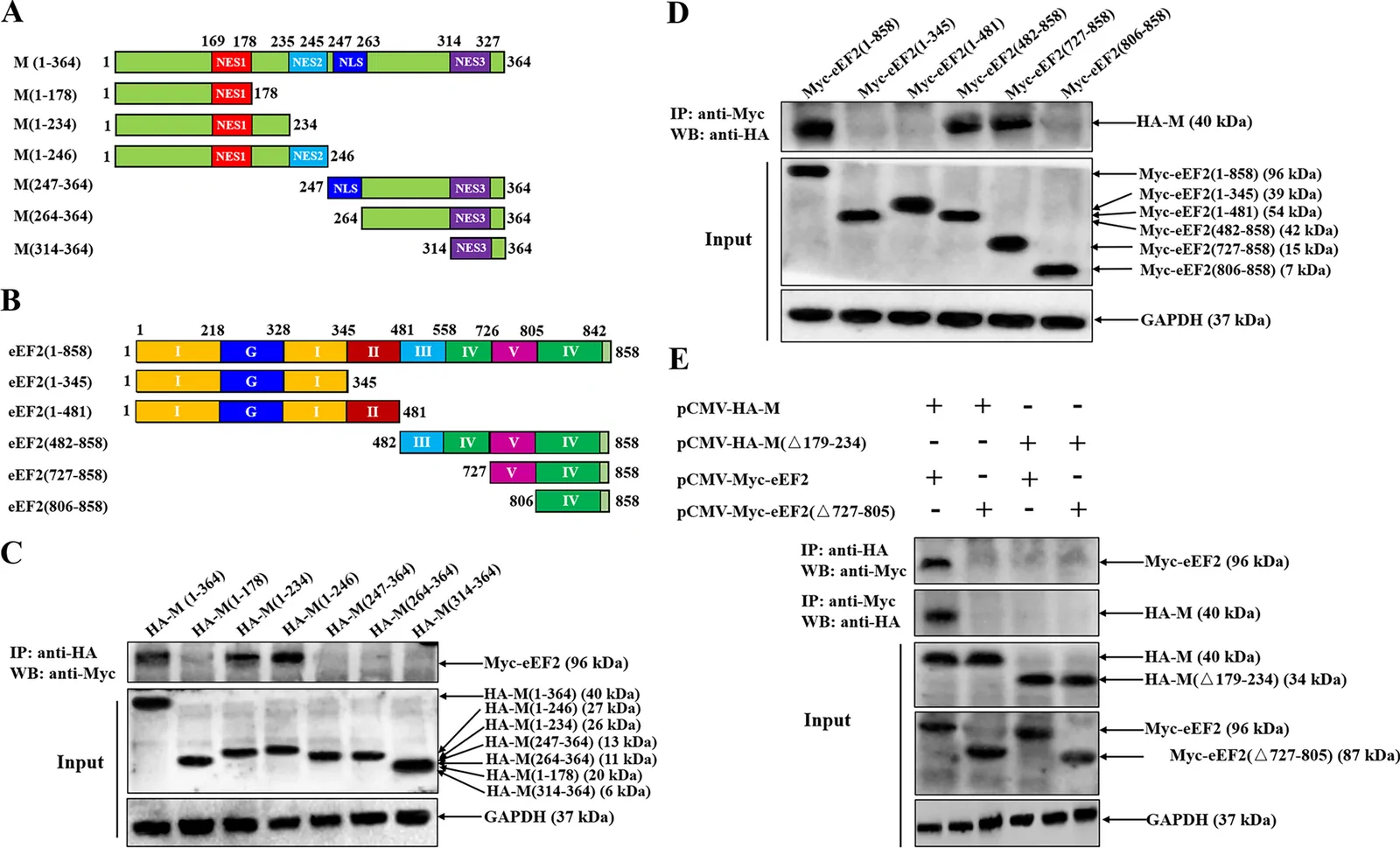
**Identification of the binding regions between NDV M protein and chicken eEF2.** Schematic representation of the full-length and truncation mutants of the NDV M protein (**A**) or chicken eEF2 (**B**). Characterization of the interaction of the full-length or truncation mutants of HA-tagged M protein or Myc-tagged chicken eEF2 with Myc-tagged chicken eEF2 (**C**) or HA-tagged M protein (**D**) by Co-IP assay, respectively. (**E**) Verification of the interaction between the HA-tagged M protein or its mutant lacking aa 179–234 and the Myc-tagged chicken eEF2 or its mutant lacking aa 727–805 by Co-IP assay.

It has been demonstrated that the NDV M protein contains one nuclear localization signal (NLS) and three nuclear export signals (NESs) that mediate its nucleocytoplasmic trafficking [34, 35], and chicken eEF2 is composed of six non-homologous domains (domain I, G, II, III, IV, and V) similar to that of prokaryotic homolog elongation factor G [36]. Therefore, a series of truncation mutants derived from the NDV M protein and chicken eEF2 were constructed to map their binding regions (Figure 2A, B). The results of Co-IP assays showed that the region (aa 179–234) located between NES1 and NES2 of the NDV M protein was important for chicken eEF2 binding, since only the HA-M(1–364), HA-M(1–234), and HA-M(1–246) fusion proteins could be immunoprecipitated with Myc-eEF2 (Figure 2C). In addition, only the fusion proteins containing the V domain (aa 727–805) of chicken eEF2, including Myc-eEF2(1–858), Myc-eEF2(482–858), and Myc-eEF2(727–858), could bind to the HA-M fusion protein (Figure 2D). Moreover, either the HA-M lacking aa 179–234 or the Myc-eEF2 lacking aa 727–805 could not interact with another full-length protein (Figure 2E). These results demonstrated that the aa 179–234 region of the NDV M protein and the V domain (aa 727–858) of chicken eEF2 were responsible for interaction with each other.

### Identification of key interactional residues between NDV M protein and chicken eEF2

**Figure 3:**
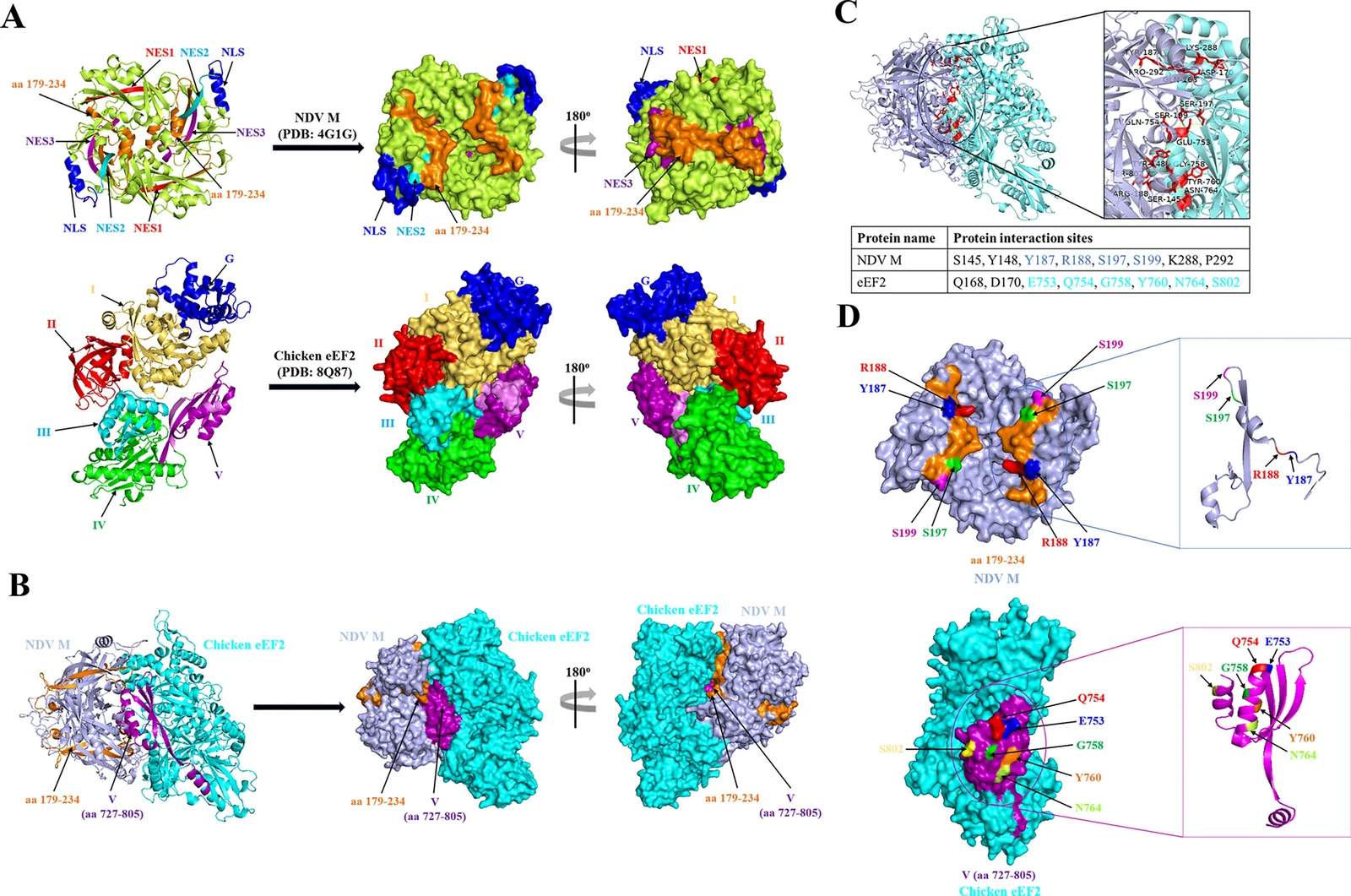
**Molecular docking and structural display of the NDV M protein and chicken eEF2.** (**A**) The three-dimensional (3D) structures of NDV M protein (PDB: 4G1G) and chicken eEF2 (PDB: 8Q87) exhibited by PyMOL 2.5.5. The known domains of the two proteins are shown in different colors, and especially, the binding regions of NDV M protein (aa 179–234) and chicken eEF2 (V domain) are marked in orange and purple, respectively. (**B**) The predicted protein complex of the NDV M protein (marked in light blue) and chicken eEF2 (marked in cyan) using AlphaFold3. The binding regions are shown in orange and purple, respectively. (**C**) Molecular docking between NDV M protein and chicken eEF2 using AlphaFold3. The interacting interfaces are highlighted within the oval box. Zoomed-in views show key residues at the interface, with hydrogen bonds and salt bridges shown in yellow. List of interactional residues at the interface is shown in the lower panel. (**D**) The 3D structures of the interactional residues in the random coil structures of NDV M protein and the α-helix structures of chicken eEF2 are labelled with distinct colors.

The binding regions between NDV M protein and chicken eEF2 were then exhibited using PyMOL 2.5.5 software. As shown in Figure 3A, the aa 179–234 region was clearly positioned between NES1 and NES2 in the cartoon drawing of the dimeric M protein and was well presented on the dimer surface of the M protein. Meanwhile, the V domain (aa 727–858) showed a relatively independent structure in the cartoon drawing of chicken eEF2, which was also exposed on the protein surface (Figure 3A). The molecular mechanism by which NDV M protein interacts with chicken eEF2 was investigated. The results of molecular docking using AlphaFold3 showed that the predicted template modeling (pTM) score was > 0.5 and the interface pTM score was > 0.8, indicating that the predicted fold of the protein complex closely resembled the true biological structure [37]. In addition, the structural display of simulated protein complex revealed that the aa 179–234 of NDV M protein and the V domain (aa 727–858) of chicken eEF2 had a close integration pattern (Figure 3B). Moreover, the predicted interactional residues were the residues S145, Y148, Y187, R188, S197, S199, K288, and P292 of M protein and the residues Q1168, D170, E753, Q754, G758, Y760, N764, and S802 of chicken eEF2, respectively (Figure 3C).

It is remarkable that four residues (Y187, R188, S197, and S199) were present in the aa 179–234 region of the NDV M protein, and six residues (E753, Q754, G758, Y760, N764, and S802) existed in the V domain (aa 727–858) of chicken eEF2 (Figure 3C), suggesting that these residues were possibly important for interaction with each other. Further structural analysis revealed that the residues Y187, R188, S197, and S199 were well presented on the dimer surface and the random coil structures of the NDV M protein, and the residues E753, Q754, G758, Y760, N764, and S802 exposed on the protein surface were all distributed in the α-helix structures of chicken eEF2 (Figure 3D).

**Figure 4:**
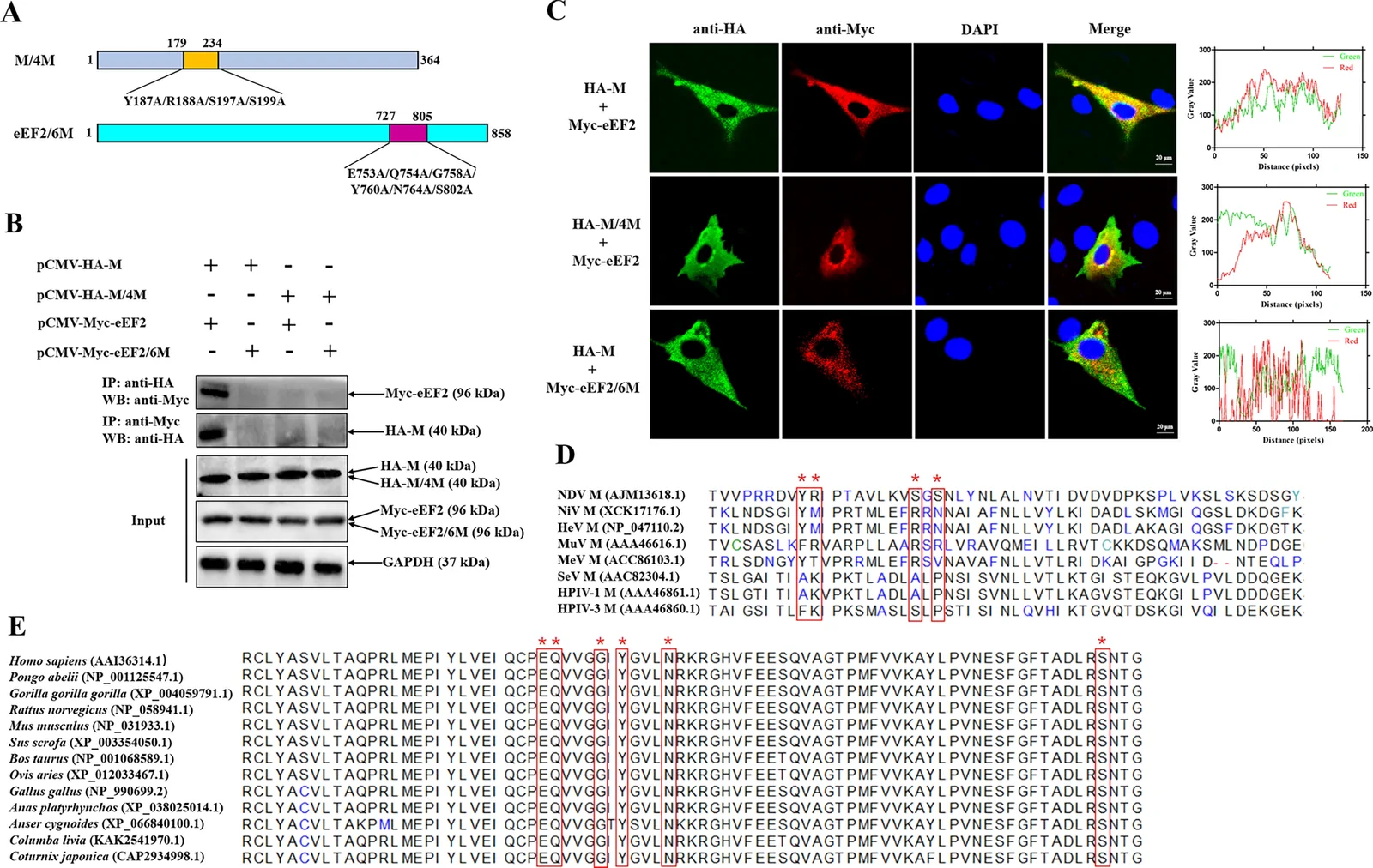
**Verification and conservation analysis of the interactional residues between NDV M protein and chicken eEF2.** (**A**) Schematic representation of Y187A/R188A/S197A/S199A and E753A/Q754A/G758A/Y760A/N764A/S802A mutations in the NDV M protein and chicken eEF2, respectively. (**B**) Identification of the interaction between HA-tagged M or M/4M and Myc-tagged eEF2 or eEF2/6M by Co-IP assay. (**C**) Fluorescence co-localization of the fusion proteins in plasmids cotransfected DF-1 cells using indirect immunofluorescence assay. The original magnification was 1 × 400. Conservation analysis of the interactional residues (marked in the red box and with an asterisk) among the M protein of NDV and other paramyxoviruses (**D**) or the eEF2 of human, other mammals, and avian species (**E**).

To further investigate whether these residues are truly responsible for M–eEF2 interaction, the mutant proteins (named as M/4M and eEF2/6M) were generated (Figure 4A). As shown in Figure 4B, no interactions were detected between the wild-type protein and another mutant protein except for the HA-M and Myc-eEF2 co-expression. Meanwhile, mutations in either M protein or chicken eEF2 decreased their fluorescence co-localizations when compared with the wild-type proteins in plasmids cotransfected cells (Figure 4C). In addition, the four residues were not conserved among NDV and other paramyxovirus M proteins (Figure 4D), while the six residues of eEF2 were highly conserved among human, other mammals, and avian species (Figure 4E). These findings indicated that the residues Y187, R188, S197, and S199 of the NDV M protein and the residues E753, Q754, G758, Y760, N764, and S802 of chicken eEF2 were essential for their binding, and this interaction might only exist in the M protein of NDV rather than other paramyxoviruses.

### Mutation of key interactional residues in the M protein attenuates NDV replication and pathogenicity

**Figure 5:**
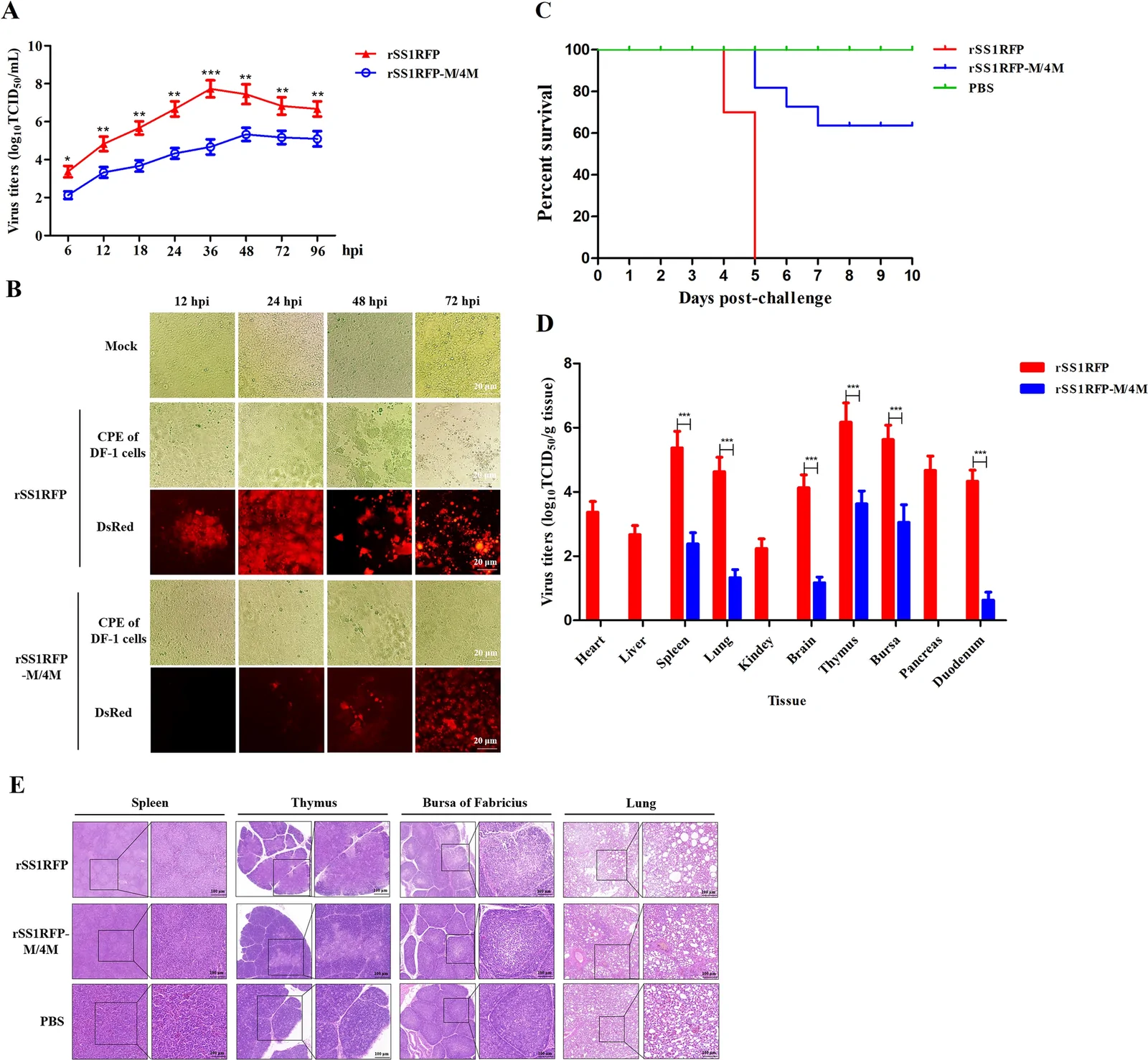
**Mutation of key interactional residues in the M protein attenuates NDV replication and pathogenicity.** (**A**) Viral titer of rSS1RFP and rSS1RFP-M/4M was determined in DF-1 cells at the indicated time points by a TCID_50_ assay. Error bars represent standard deviations (mean ± SD) (^*^P < 0.05; ^**^P < 0.01; ^***^P < 0.001 compared with the viral titer of rSS1RFP-M/4M). (**B**) The CPE and DsRed expression were observed in rSS1RFP- and rSS1RFP-M/4M-infected DF-1 cells at the indicated time points. The original magnification was 1 × 200. (**C**) Survival curves of 4-week-old SPF chickens inoculated with rSS1RFP and rSS1RFP-M/4M at a dose of 10^5.0^ EID_50_/100 μL per bird or with 100 μL PBS as the negative control. The birds were monitored for mortality rate statistics daily for 10 dpi. (**D**) Viral load in the collected tissues of 4-week-old chickens infected with rSS1RFP and rSS1RFP-M/4M at 4 dpi. Viral titers were determined in DF-1 cells and presented as log_10_ TCID_50_ g^−1^ tissue. Error bars represent standard deviations (mean ± SD) (^***^P < 0.001 compared with the virus titer of rSS1RFP-M/4M). (**E**) Histopathology of lymphoid tissue and lung tissue samples collected from rSS1RFP- and rSS1RFP-M/4M- or PBS-inoculated 4-week-old chickens.

To explore the effect of key interactional residues Y187, R188, S197, and S199 in the M protein that bind to chicken eEF2 on the replication and pathogenicity of NDV, the mutant virus rSS1RFP-M/4M harboring Y187A/R188A/S197A/S199A in the M protein was rescued by reverse genetic manipulation. Whole-genome sequence analysis of rSS1RFP-M/4M after five passages showed that no additional mutations were observed in the *M* gene and other viral genes besides the artificially introduced mutations. Thereafter, the biological characteristics of the parental NDV (rSS1RFP) and mutant NDV (rSS1RFP-M/4M) was examined. As shown in Table 3, the MDT of rSS1RFP-M/4M (102 ± 2 h) was obviously delayed in embryonated chicken eggs when compared with that of rSS1RFP (54 ± 2 h), and the ICPI, EID_50_, TCID_50_, and HA values of rSS1RFP-M/4M were also much lower than that of rSS1RFP, indicating that these residue mutations could lead to the decreased virulence and weakened replication ability of NDV. In addition, the results of multicycle growth kinetics showed that the viral titers of rSS1RFP-M/4M were significantly decreased in comparison to that of rSS1RFP from 12 to 96 hpi (*P* < 0.01) (Figure 5A). Moreover, the prominent CPE and DsRed expression were observed in rSS1RFP-infected cells at 12 hpi and cell monolayer was absolutely destroyed at 72 hpi; on the contrary, the slight CPE and DsRed expression in rSS1RFP-M/4M-infected cells started at 24 hpi, and cell monolayers were still existent in large quantities at 72 hpi (Figure 5B).

**Table 3:** **Biological characteristics of the parental and mutant NDVs**

Virus	Pathogenicity^*^		Virus titer^**^		
	MDT (h)	ICPI	EID_50_/mL	TCID_50_/mL	HA
rSS1RFP	54 ± 2	1.88 ± 0.04	10^8.67^	10^8.50^	7 log_2_
rSS1RFP-M/4M	102 ± 2	1.36 ± 0.05	10^5.13^	10^4.75^	4 log_2_

^*^MDT mean death time, *ICPI* intracerebral pathogenicity index, ^**^EID_*50*_ 50% egg infectious dose, *TCID*_*50*_ 50% tissue culture infective dose.

The in vivo pathogenicity assessment of NDV in 4-week-old SPF chickens was then performed. The results of survival curves indicated that chickens inoculated with the parental virus rSS1RFP exhibited 100% mortality by 5 dpi, but the mutant virus rSS1RFP-M/4M only caused 40% mortality by 7 dpi, and there was no death in the subsequent days (Figure 5C). Meanwhile, Virus titration analysis showed that rSS1RFP-M/4M only had weak replication ability in the tissues of spleen, lung, brain, thymus, bursa, and duodenum at 4 dpi, whereas rSS1RFP could replicate in multiple tissues and had relatively higher virus titers (Figure 5D). Additionally, rSS1RFP infection leaded to multifocal confluent coagulative necrosis, severe lymphocyte depletion, infiltration of macrophages in the lymphoid tissues (spleen, thymus, and bursa), and extreme expansion in the bronchi of the lung (Figure 5E). By contrast, rSS1RFP-M/4M infection merely occurred with slight histopathological changes in the above tissues, and as expected, no apparent histopathological changes were observed in the PBS inoculation group (Figure 5E). Thus, these data suggested that mutation of key interactional residues (Y187A/R188A/S197A/S199A) in the M protein losing its binding ability to chicken eEF2 significantly attenuated the replication and pathogenicity of NDV.

### NDV M protein upregulates chicken eEF2 expression to promote viral protein production and enhance viral particle yield

**Figure 6:**
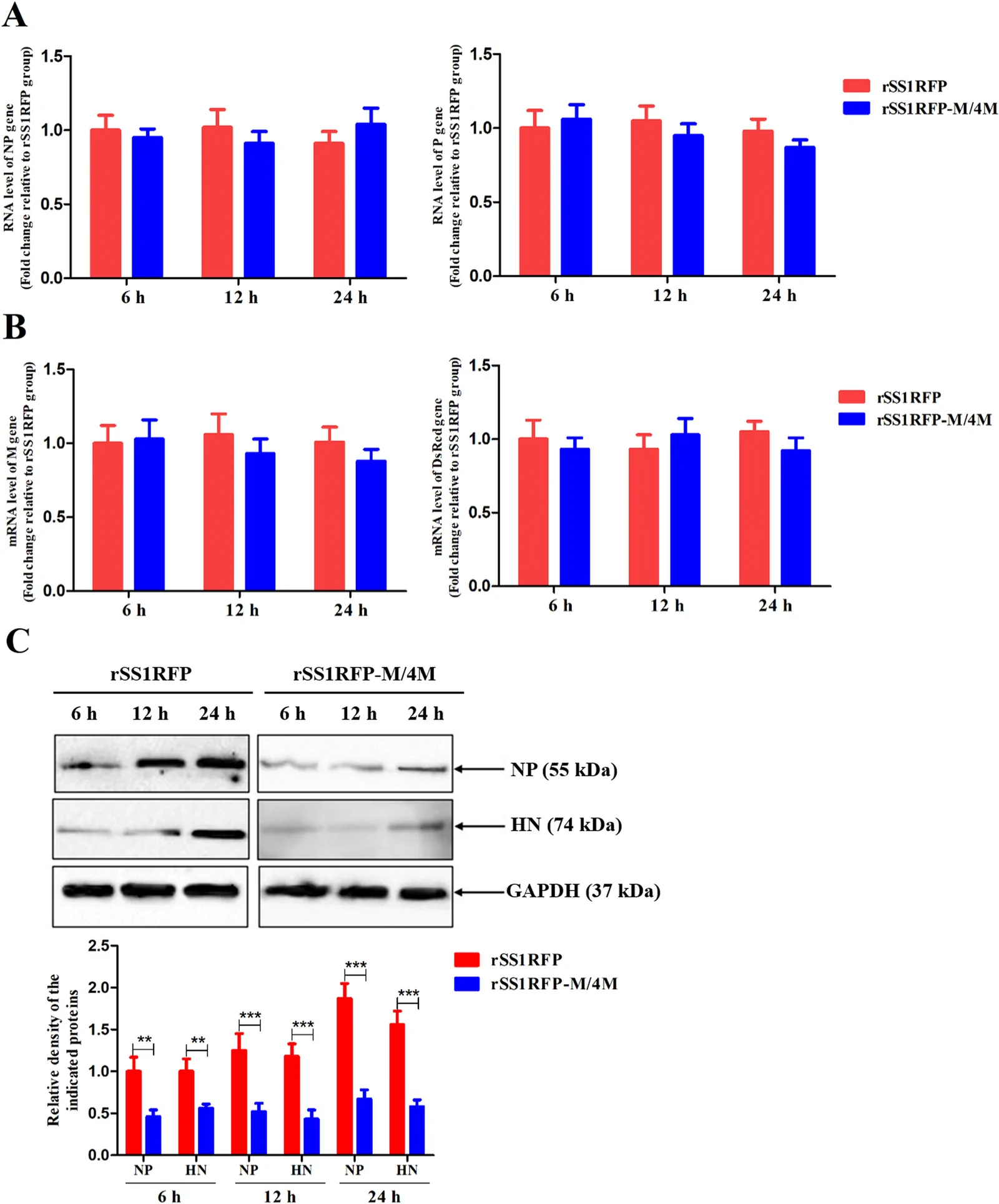
**The effect of key interactional residues mutation in the M protein on the vRNA synthesis, transcription, and translation of NDV.** (**A**) The vRNA synthesis corresponding to the *NP* and *P* genes and (**B**) viral transcription corresponding to the *M* and *DsRed* genes in rSS1RFP- and rSS1RFP-M/4M-infected DF-1 cells were examined by qRT-PCR at the indicated time points. Error bars represent standard deviations (mean ± SD) (no asterisk indicates no significant difference). (**C**) The expression levels of viral NP and HN proteins in rSS1RFP- and rSS1RFP-M/4M-infected DF-1 cells were detected by western blot at the indicated time points. The relative levels of the viral NP and HN proteins were compared with the control GAPDH expression. Error bars represent standard deviations (mean ± SD) (^**^P < 0.01;^***^P < 0.001 compared with the viral protein expression of rSS1RFP-M/4M).

To further investigate how Y187A/R188A/S197A/S199A mutations in the M protein affects NDV replication and pathogenicity, the vRNA synthesis, transcription, and translation of rSS1RFP and rSS1RFP-M/4M were firstly explored. As shown in Figure 6A and B, the relative vRNA levels (corresponding to the *NP* and *P* genes) and viral mRNA levels (corresponding to the *M* and *DsRed* genes) between rSS1RFP- and rSS1RFP-M/4M-infected cells had no statistically significant differences at 6, 12, and 24 hpi. However, there was more decreased expression levels of the viral NP and HN proteins of rSS1RFP-M/4M than those of rSS1RFP at 6 hpi (*P* < 0.01), and the expression levels of these viral proteins were much more significant between rSS1RFP-M/4M and rSS1RFP at 12 and 24 hpi (*P* < 0.001) (Figure 6C), indicating that the decreased expression of viral proteins was associated with the mutation of Y187, R188, S197, and S199 residues in the NDV M protein.

**Figure 7:**
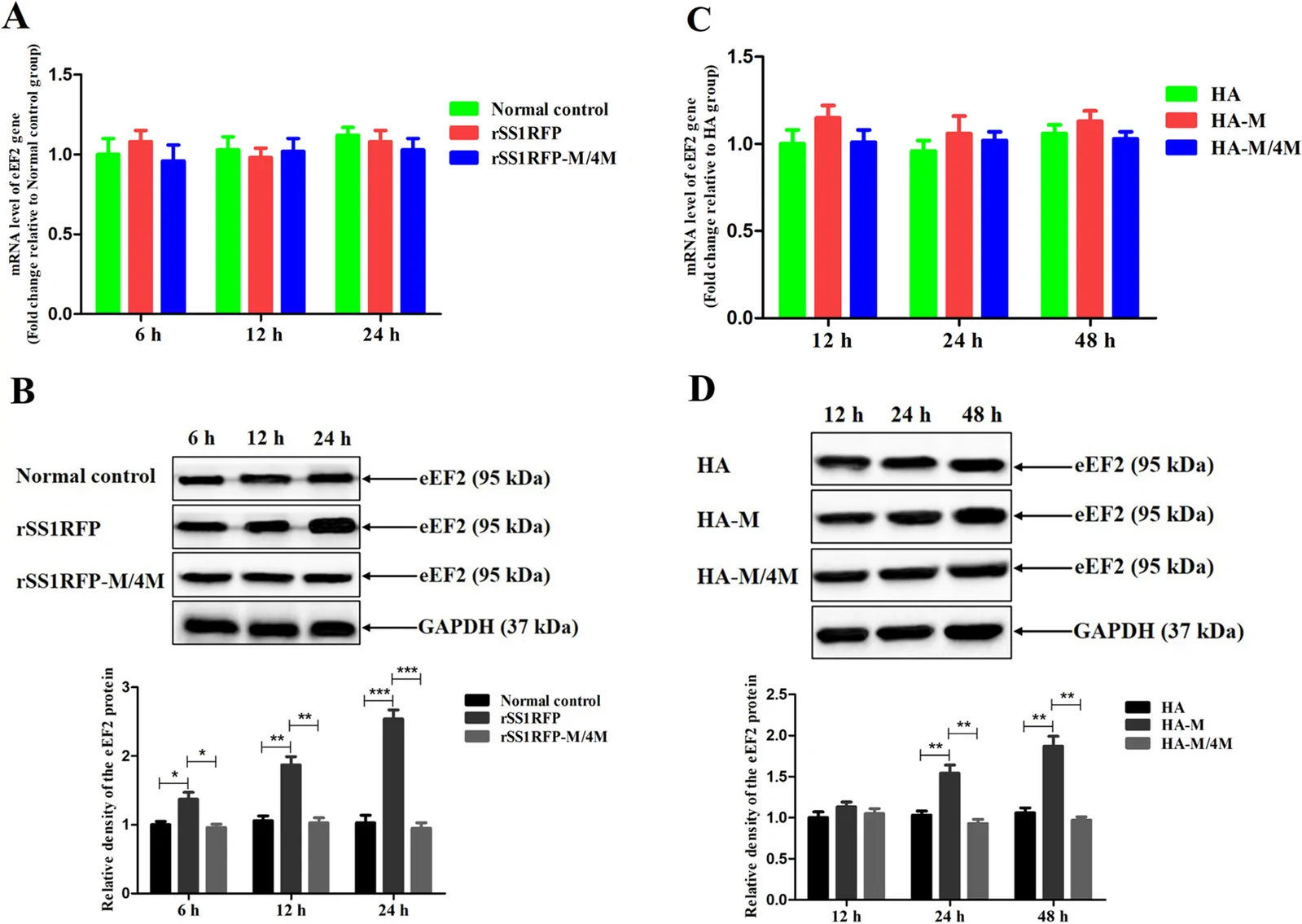
**The effect of NDV infection or M protein overexpression on the transcription and translation levels of chicken eEF2.** The transcription and translation levels of chicken eEF2 in rSS1RFP- and rSS1RFP-M/4M-infected DF-1 cells (**A** and **B**) or in HA-M- and HA-M/4M-overexpressed DF-1 cells (**C** and **D**) were detected by qRT-PCR and western blot at the indicated time points, respectively. Error bars represent standard deviations (mean ± SD) (^*^P < 0.05; ^**^P < 0.01; ^***^P < 0.001 compared with chicken eEF2 of the rSS1RFP-M/4M group or HA-M/4M group; no asterisk indicates no significant difference).

The effect of NDV infection or M protein overexpression on the transcription and translation levels of chicken eEF2, as well as the effect of chicken eEF2 knockdown or overexpression on the viral budding efficiency, viral titer, and cytopathogenicity of NDV were then evaluated. It was found that the transcription levels of the chicken *eEF2* gene were not altered in both rSS1RFP- and rSS1RFP-M/4M-infected cells (Figure 7A); however, rSS1RFP rather than rSS1RFP-M/4M infection upregulated the expression level of chicken eEF2 at 6, 12, and 24 hpi (Figure 7B). Similarly, the translation level but not the transcription level of chicken eEF2 was obviously increased in the HA-M overexpression group than that in the HA and HA-M/4M overexpression groups at 24 and 48 hpt (Figure 7C, D), suggesting that the existence of NDV M protein increased the translation level of chicken eEF2.

**Figure 8:**
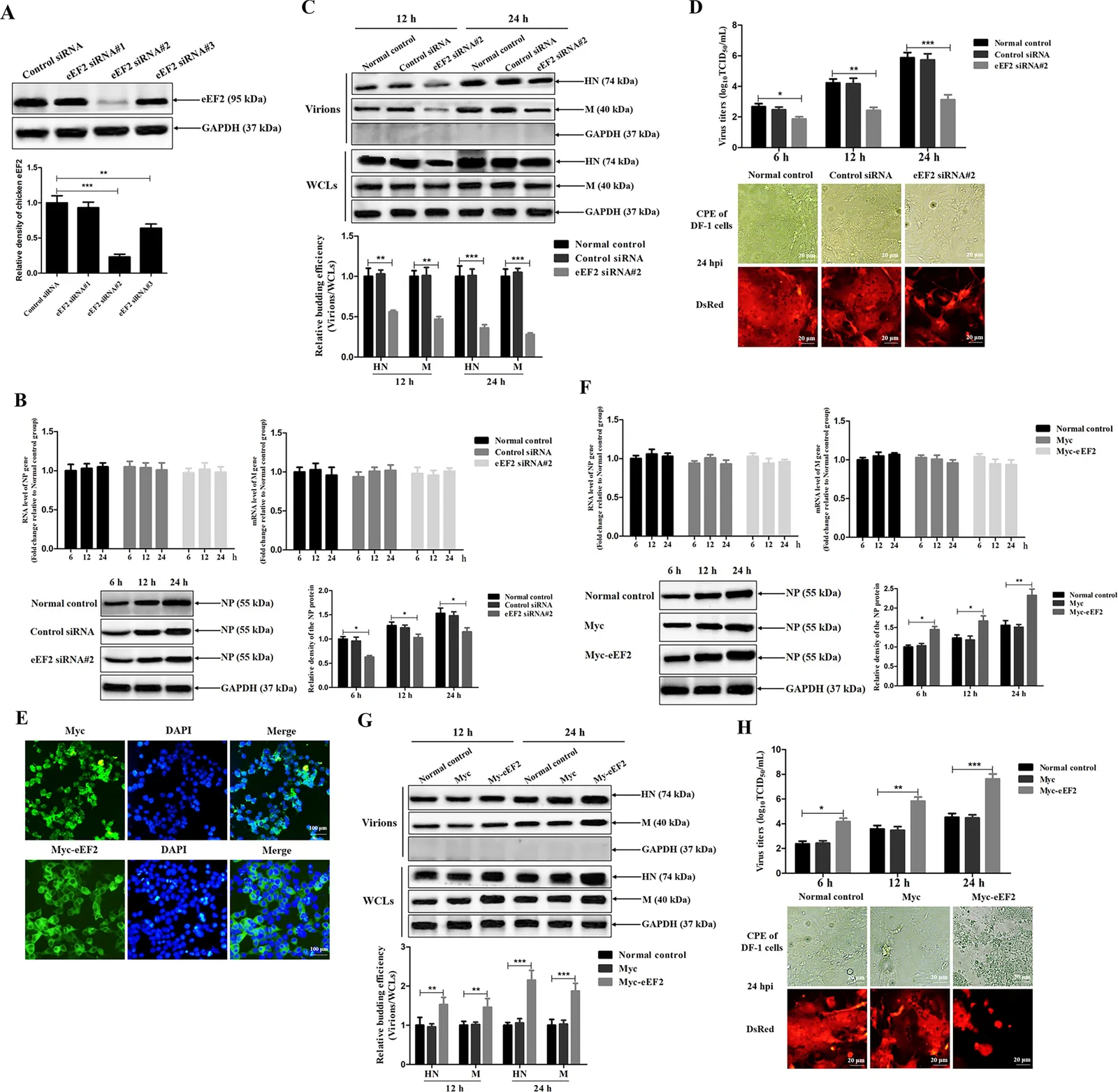
**Knockdown or overexpression of chicken eEF2 affects viral protein production and viral particle yield.** (**A**) Detection of chicken eEF2 siRNAs or control siRNA transfection on the expression level of chicken eEF2 in DF-1 cells by western blot. Error bars represent standard deviations (mean ± SD) (^**^P < 0.01; ^***^P < 0.001 compared with control siRNA group). The effect of eEF2 siRNA#2-mediated knockdown of chicken eEF2 on the vRNA synthesis, transcription, and translation (**B**), and viral budding efficiency (**C**), as well as viral titer, CPE, and DsRed expression (**D**) of rSS1RFP. Error bars represent standard deviations (mean ± SD) (^*^P < 0.05; ^**^P < 0.01 compared with normal control group). (**E**) Fluorescence observation of Myc and Myc-eEF2 in plasmid-transfected DF-1 cells. The original magnification was 1 × 200. The effect of Myc or Myc-eEF2 overexpression on the vRNA synthesis, transcription, and translation (**F**), and viral budding efficiency (**G**), as well as viral titer, CPE, and DsRed expression (**H**) of rSS1RFP. Error bars represent standard deviations (mean ± SD) (^*^P < 0.05; ^**^P < 0.01 compared with normal control group).

Afterward, three pairs of chicken eEF2 siRNAs (Table 2) were transfected into DF-1 cells, and eEF2 siRNA#2 could more effectively reduce chicken eEF2 expression than other eEF2 siRNAs and the control siRNA (Figure 8A). Meanwhile, eEF2 siRNA#2-mediated knockdown of chicken eEF2 did not affect the vRNA synthesis and transcription of rSS1RFP but reduced viral protein production (Figure 8B). Consistent with this finding, the viral budding efficiency of the eEF2 siRNA#2 group was obviously lower than that of the normal control and control siRNA groups at 12 and 24 hpi (Figure 8C). Meanwhile, the decreased viral titer, cytopathogenicity, and DsRed expression of rSS1RFP was also observed in the eEF2 siRNA#2 group (Figure 8D). On the contrary, overexpression of Myc-eEF2 rather than Myc tag promoted the expression of viral proteins, while the vRNA synthesis and transcription of rSS1RFP were not changed among Myc- and Myc-eEF2-overexpression and normal control groups (Figure 8E and F). Importantly, overexpression of Myc-eEF2 significantly enhanced the budding efficiency, viral titer, and cytopathogenicity of rSS1RFP when compared with that of normal control and Myc-overexpression groups (Figure 8G, H). Overall, these results indicated that the NDV M protein was responsible for the increased expression of chicken eEF2, and its elevated expression level could promote viral protein production and viral budding efficiency and viral titer, which was conducive to viral particle yield and the replication and pathogenicity of NDV.

### NDV M protein protects chicken eEF2 from phosphorylation to increase viral protein production and viral replication

**Figure 9:**
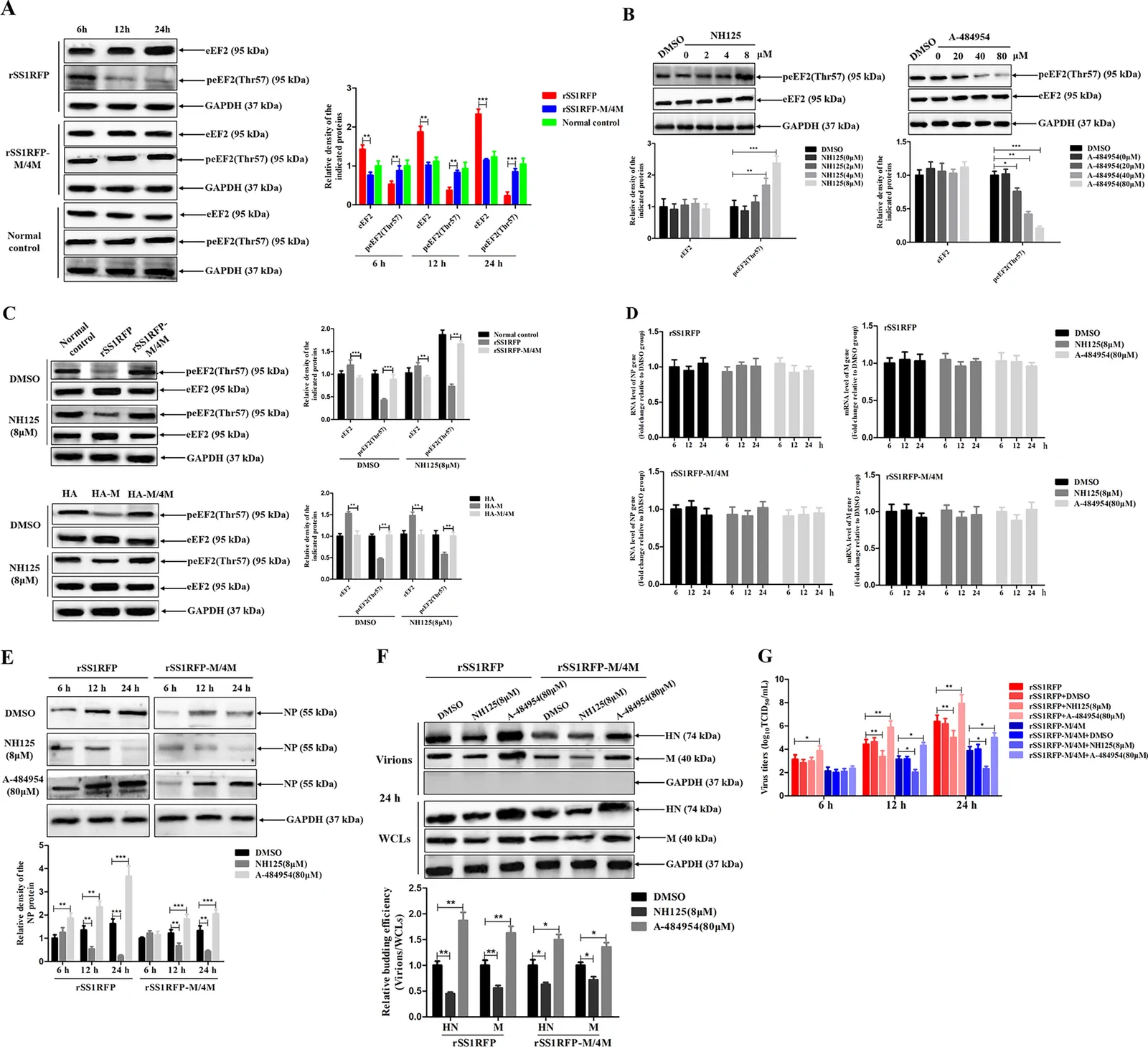
**NDV M protein protects chicken eEF2 from phosphorylation to increase viral protein production and viral replication.** (**A**) The expression levels of chicken eEF2 and peEF2 (Thr57) in rSS1RFP- and rSS1RFP-M/4M-infected DF-1 cells were detected at the indicated time points by western blot. Error bars represent standard deviations (mean ± SD) (^**^P < 0.01; ^***^P < 0.001 compared with normal control group). (**B**) Different concentrations of NH125 (0, 2, 4, 8, and 12 μM) and A-484954 (0, 20, 40, 80, and 120 μM) treatment on the expression of chicken eEF2 and peEF2 (Thr57) in DF-1 cells. Error bars represent standard deviations (mean ± SD) (^*^P < 0.05;^**^P < 0.01;^***^P < 0.001 compared with DMSO group). (**C**) NH125 (8 μM) treatment on the expression of chicken eEF2 and peEF2 (Thr57) in rSS1RFP- and rSS1RFP-M/4M-infected or HA-M and HA-M/4M-overexpressed DF-1 cells. Error bars represent standard deviations (mean ± SD) (^**^P < 0.01;^***^P < 0.001 compared with DMSO group). The effect of NH125 (8 μM) or A-484954 (80 μM) treatment on the vRNA synthesis and transcription (**D**), viral protein expression (**E**), viral budding efficiency (**F**), and viral titer (**G**) of rSS1RFP and rSS1RFP-M/4M. Error bars represent standard deviations (mean ± SD) (^*^P < 0.05; ^**^P < 0.01^***^P < 0.001 compared with DMSO group; no asterisk indicates no significant difference).

Studies have shown that eEF2-occurring phosphorylation at Thr57 residue can lead to inactivation of eEF2 and inhibition of subsequent cellular or viral protein synthesis rate [25, 38, 39], so the relationship between the peEF2 (Thr57) level and NDV replication was further investigated. As shown in Figure 9A, the expression levels of chicken eEF2 were gradually increased during rSS1RFP infection from 6 to 24 hpi when compared with rSS1RFP-M/4M and normal control groups, which was consistent with the results found in Figure 7B and D. However, the peEF2 (Thr57) levels were oppositely decreased in rSS1RFP- rather than rSS1RFP-M/4M-infected cells at 6, 12, and 24 hpi, respectively (Figure 9A). It has been shown that eEF2 kinase (eEF2K) is the only kinase known to phosphorylate eEF2, while chemical compounds NH125 and A-484954 are the highly selective eEF2K inhibitors, which specifically increase or reduce the peEF2 (Thr57) levels with little effect on cell growth, respectively [40]. Here, DF-1 cells treated with different concentrations of chemical compounds showed a significant increase or decrease in the peEF2 (Thr57) expression levels accompanied by the increased concentrations of NH125 and A-484954, respectively (Figure 9B). Meanwhile, NH125 and A-484954 with concentrations of 8 μM and 80 μM, respectively, had the most obvious effect on the increased or decreased levels of peEF2 (Thr57) (Figure 9B), which had little effect on proliferation in DF-1 cells (Additional file 2).

Next, the effect of NH125 (8 μM) treatment on the expression levels of chicken eEF2 and peEF2 (Thr57) in NDV-infected or plasmid-transfected DF-1 cells was investigated. The results revealed that the expression levels of eEF2 and peEF2 (Thr57) in rSS1RFP-infected or pCMV-HA-M-transfected cells were much higher or lower than that in rSS1RFP-M/4M-infected and pCMV-HA-M/4M-transfected cells when treated with either DMSO or NH125 (8 μM), respectively (Figure 9C), suggesting that the existence of the NDV M protein reduced the peEF2 (Thr57) expression level. Remarkably, DF-1 cells treated with DMSO, NH125 (8 μM), or A-484954 (80 μM) had no effect on the vRNA synthesis and transcription of rSS1RFP and rSS1RFP-M/4M (Figure 9D), while the production of viral NP protein showed an obvious decrease or increase level in rSS1RFP- and rSS1RFP-M/4M-infected cells when treated with NH125 (8 μM) and A-484954 (80 μM), respectively (Figure 9E). Additionally, it is just as expected that the viral budding efficiency and viral titer of rSS1RFP and rSS1RFP-M/4M showed the decreased or increased patterns in cells treated with NH125 (8 μM) or A-484954 (80 μM) (Figure 9F, G), which were correlated well with the changed levels of peEF2 (Thr57). Therefore, these results suggested that the binding of M protein to chicken eEF2 protected it from phosphorylation to potentiate viral protein production, viral budding efficiency, and viral titer, as well as the subsequent viral replication process.

## Discussion

NDV strictly depends on host cells to complete its life cycle; therefore, exploring virus–host interactions is considerable for revealing the underlying replication and pathogenesis of NDV [12]. Currently, research on viral protein production has mainly focused on the interaction of NDV NP protein with host proteins. For instance, NDV infection activates the PI3K/Akt/mTOR and p38 MAPK/Mnk1 pathways to benefit selective cap-dependent translation of viral mRNAs via the interaction of NP protein with host eIF4E [41]. Meanwhile, the NDV NP protein can also interact with host eIF4A1 to inhibit cellular mRNAs translation and enhance viral mRNAs translation efficiency [42]. However, whether other NDV proteins are involved in viral protein production still remains unclear. It is notable that the M protein of several paramyxoviruses, including human parainfluenza virus 3 (HPIV3) and Nipah virus (NiV), can redirect ribosomes to translate viral mRNAs at the expense of host cell transcripts; meanwhile, the interaction of viral M protein with host cEJC further inhibits host cells’ nascent peptide synthesis and boosts the translation efficiency of viral mRNAs [19]. We previously found that the association of RPL18 with NDV M protein promotes viral mRNA translation and viral replication [20]. Therefore, these insights highlight a new strategy on how the M protein exploits host proteins to facilitate the protein production of paramyxoviruses, including NDV. Nevertheless, more studies are required to investigate whether there are other host proteins interacting with paramyxovirus M proteins to regulate the production of viral proteins.

As a member of the G-protein superfamily, eEF2 is highly conserved among mammalian and avian species, which regulates the process of translation elongation and protein synthesis [43]. In our and other previous studies, chicken eEF2 was identified as a new binding partner with the NDV M protein [15, 28, 29], but the actual interaction of M protein with chicken eEF2 and the role of this interaction in the replication and pathogenicity of NDV are not clear. In this study, the NDV M protein was confirmed to directly interact with chicken eEF2 in the cytoplasm, as evidenced by the results of fluorescence co-localization, Co-IP, and pull-down assays. In addition, the N-terminus region (aa 179–234) of NDV M protein and the V domain (aa 727–858) of chicken eEF2 were critical for their interaction. It is reported that the domain V together with domains III and IV comprise the second super-domain of eEF2, which play central roles in anchoring mRNAs to the ribosome, assisting conformational switch of the ribosome, transmitting allosteric signals of eEF2, and supporting translocation completion of tRNA [44–46]. Therefore, it can be hypothesized that the binding of M protein to the V domain of chicken eEF2 may contribute to NDV-induced shut-off of host translation and the priority translation of viral mRNAs. However, the detailed mechanism by which the NDV M protein hijacks chicken eEF2 to regulate host and viral translation remains to be explored.

The identification of key interactional residues is of great significance for deeply understanding the potential function of viral proteins. Here, the results of molecular docking and structure display showed that four residues (Y187, R188, S197, and S199) located at the N-terminus region (aa 179–234) of NDV M protein and six residues (E753, Q754, G758, Y760, N764, and S802) distributed in the V domain of chicken eEF2 were responsible for their binding. The importance of this binding in M-eEF2 interaction was subsequently verified by Co-IP and fluorescence co-localization assays. It is noteworthy that all the four residues mainly concentrated in the random coil structures of the NDV M protein, and the six residues were predominantly presented on the α-helix structures of chicken eEF2, which might be conducive to the binding and dissociation of the M–eEF2 complex [47]. However, conservation analysis of these key interactional residues indicated that six residues (E753, Q754, G758, Y760, N764, and S802) of eEF2 were highly conserved among human, other mammals, and avian species, while four residues (Y187, R188, S197, and S199) of M protein were completely nonconservative between NDV and other paramyxoviruses. This finding suggests that the M–eEF2 interaction possibly plays a unique role in the NDV life cycle.

It is well-known that the NDV M protein is a highly basic and hydrophobic protein, which contains a large number of basic (e.g., R, K, and H), hydrophobic (e.g., I, L, and V), and polar neutral (e.g., S, T, and Y) aa residues [48]. Previous studies have mainly focused on the functions of basic or hydrophobic residues in the NLS and NES of the NDV M protein that determine its nucleocytoplasmic trafficking [6, 7, 15, 18, 32]. Meanwhile, our recent studies also found that mutation of three basic residues (R283A/R286A/K288A) in the C-terminus of M protein attenuates the replication and pathogenicity of NDV by reducing vRNA synthesis and transcription [49]. However, the functions of other residues in the NDV M protein are poorly understood. In the present study, the mutant virus rSS1RFP-M/4M harboring Y187A/R188A/S197A/S199A in the M protein was successfully rescued. These key residue mutations rendered the M protein incapable of binding to chicken eEF2, which remarkably attenuated the virulence, replication ability, and pathogenicity of NDV. More importantly, different from the previous findings, our results demonstrated that this mutation predominantly reduced viral protein production rather than affected vRNA synthesis and transcription, which is consistent with the latest finding that paramyxovirus M protein enhances viral protein production without affecting viral mRNA transcription [19].

Currently, the viral proteins known to undergo phosphorylation modification are mainly the paramyxovirus P protein [31, 50–52], while the most frequently reported post-translational modification of paramyxovirus M protein is the M’s ubiquitination-mediated viral budding and release processes [6, 53–55]. Although the paramyxovirus M proteins contain abundant S, T, and Y residues, little is known about whether these residues cause phosphorylation modification and their specific functions. Previous studies have shown that the phosphorylation of S70 residue in Sendai virus M protein is not essential for viral replication either in vitro or in vivo [56]. However, another study revealed that a portion of M protein within parainfluenza virus 5-infected cells is phosphorylated at S369 residue, which enhances its interaction with host protein 14-3-3 to negatively regulate viral particle formation [57]. Therefore, there is no consensus on whether the phosphorylation modification of paramyxovirus M protein is related to viral replication. One recent study used IP–mass spectrometry to identify the phosphorylation modification sites of NDV M protein and found that Y187 is one of the 14 potential phosphorylation sites of the M protein [58]. Although it is still uncertain whether Y187 is truly phosphorylated, based on our current research results, it can be determined that Y187 together with the other three residues (R188, S197, and S199) are crucial for the interaction between NDV M protein and chicken eEF2 as well as for the replication and pathogenicity of NDV. Therefore, these findings will undoubtedly provide a deeper understanding of the function of basic (R) and polar neutral (Y and S) residues in the NDV M protein.

Our research further revealed that the NDV M protein interacted with chicken eEF2 to upregulate its expression level and reduce its phosphorylation level, which promoted viral protein production and enhanced viral budding efficiency and viral titer. Therefore, it can be concluded that NDV M–eEF2 interaction plays an important role in the process of viral budding and release, which are helpful in increasing viral particle yield and the subsequent viral replication and pathogenicity. It has been shown that the decreased level of peEF2 (Thr57) activates eEF2 to facilitate translation elongation and protein synthesis [23, 25, 59]. However, it is interesting that the M protein entered the cytoplasm and bound to chicken eEF2 only at the late stage of NDV infection, but the peEF2 (Thr57) level had already decreased in the early stage of NDV infection. In fact, our previous study has demonstrated that multiple signaling cascades, including p38 MAPK, mTORC1/p70 S6K, and ERK1/2/p90 RSK1, are activated to regulate the phosphorylation levels and activities of eEF2K and eEF2 early in NDV infection, which contributes to the enhanced viral protein production and viral replication [60]. Because there is evidence suggesting that early NDV infection has almost completely abolished host de novo protein synthesis through manipulating various cellular signaling pathways [41, 61–63], it is speculated that the early decreased level of peEF2 (Thr57) during NDV infection may be utilized for promoting the translation efficiency of viral mRNAs, and the further reduced level of peEF2 (Thr57) caused by the cytoplasmic M protein is more conducive to the production of viral proteins and viral particles. However, these speculations require more experimental evidence, such as polysome profile analysis, translation inhibition assay, integrated transcriptome-wide ribosome profiling, etc., to be elucidated in future studies.

**Figure 10:**
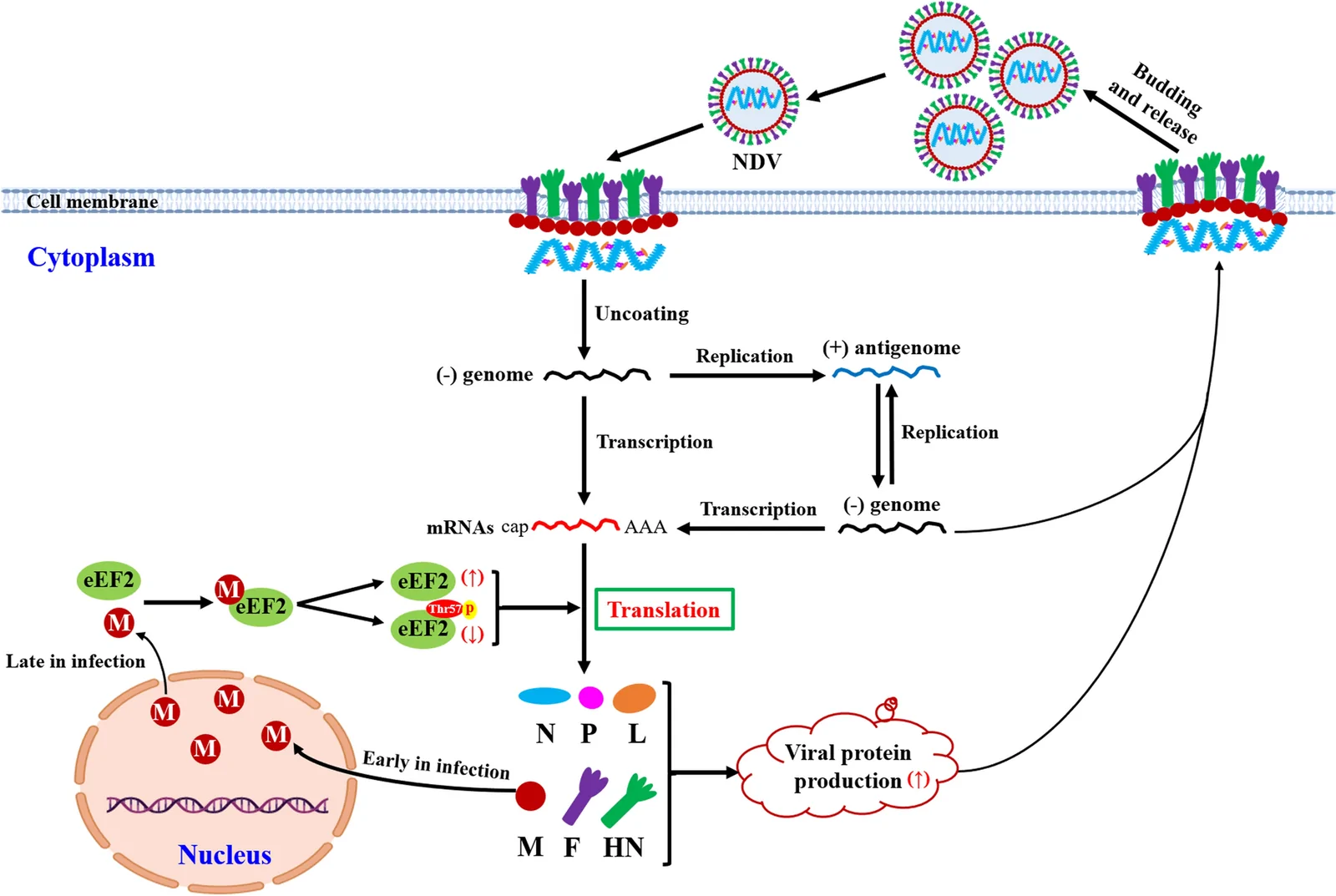
**Schematic diagram of the mechanism by which the M protein interacts with chicken eEF2 to enhance NDV replication and pathogenicity.** The M protein undergoes the nucleocytoplasmic trafficking during NDV infection, which principally interacts with chicken eEF2 in the cytoplasm late in virus infection. The M–eEF2 interaction not only increases the translation level of chicken eEF2 but also prevents its phosphorylation to reduce the peEF2 (Thr57) level, which jointly promotes the production of viral proteins and the budding and release of progeny virions and thereby enhances viral particle yield to facilitate the replication and pathogenicity of NDV.

In conclusion, this study demonstrates the biological significance of M–eEF2 interaction in the replication and pathogenicity of NDV. In terms of the mechanism, on one hand, the M protein enters the cytoplasm late in NDV infection to interact with chicken eEF2 and increase its expression at the translation level, and on the other hand, the binding of M protein to chicken eEF2 prevents its phosphorylation to reduce the peEF2 (Thr57) level, which work together to promote the production of viral proteins and the budding and release of progeny virions, thereby enhancing viral particle yield to facilitate the replication and pathogenicity of NDV (Figure 10). Collectively, these findings provide direct evidence supporting the NDV M protein involved in the viral protein production and offer insights into the development of novel anti-NDV drugs through targeting chicken eEF2.

## Supplementary information

**Additional file 1. Construction of the full-length cDNA clone pNDV/SS1RFP-M/4M harboring four alanine (A) substitutions in the M protein (Y187A/R188A/S197A/S199A). **The fragment containing the restriction enzyme sties *Bsh*TⅠ and *Bst*1107Ⅰ in the plasmid pNDV/SS1RFP was ampliﬁed by two pairs of speciﬁc primers (M/4M-F and M/4M-a, M/4M-b and M/4M-R), which were used to introduce four A substitutions (Y187A/R188A/S197A/S199A) in the M protein, to generate two PCR fragments. The two fragments were joined in a second overlapping PCR using the primers M/4M-F and M/4M-R. The newly generated fragment was used to replace the corresponding region in the plasmid pNDV/SS1RFP by enzymatic digestion, ligation and transformation methods. The resulting full-length cDNA clone was named as pNDV/SS1RFP-M/4M.

**Additional file 2. The effect of different concentrations of NH125 and A-484954 treatment on the proliferation of DF-1 cells**. DF-1 cells were treated with NH125 (0, 2, 4, 8 and 12 μM) (**A**) or A-484954 (0, 20, 40, 80 and 120 μM) (**C**) for 24 h, and the enhanced cell counting kit-8 was used to determine cell viability at 450 nm wavelength using a microplate reader. (**B and D**) Cell proliferation of NH125- or A-484954-treated DF-1 cells was assessed using Ki67 assay. Error bars represent standard deviations (mean ± SD) (**P< 0.01 compared to DMSO group).

## Data Availability

The datasets used and/or analyzed during the current study are available from the corresponding author on reasonable request.
